# Employee Voice: A Mechanism to Harness Employees’ Potential for Sustainable Success

**DOI:** 10.3390/ijerph19020921

**Published:** 2022-01-14

**Authors:** Hengwei Zhu, Muhammad Kamran Khan, Shakira Nazeer, Li Li, Qinghua Fu, Daniel Badulescu, Alina Badulescu

**Affiliations:** 1 Art School, City College of Huizhou, Huizhou 516000, China; zhu8872428@163.com; 2 Department of Management Sciences, Virtual University of Pakistan, Lahore 54000, Pakistan; rmkamrankhan@gmail.com (M.K.K.); Shakira.nazeer@vu.edu.pk (S.N.); 3 Design Academy Sichuan Fine Arts Institute, Wuhan 430072, China; 4 Department of Business Administration, Moutai Institute, Zunyi 563000, China; 5 Department of Economics and Business, Faculty of Economic Sciences, University of Oradea, 410087 Oradea, Romania; dbadulescu@uoradea.ro (D.B.); abadulescu@uoradea.ro (A.B.)

**Keywords:** ethical leadership (EL), psychological safety (PS), voice behavior (VB), leader–member exchange (LMX), oil and gas sector (O&G), petroleum industry

## Abstract

Listening to employees’ concerns reduces their dissatisfaction, but moreover, for an organization to achieve sustainable success, employees must raise their creative voice and give their input in decision-making without the fear of rejection in a psychologically safe environment. Ethical leaders facilitate such a participative style of management. A bureaucratic culture, as is generally encountered in Pakistan’s work settings, poses real challenges to those who dare to speak up, therefore the importance of ethical leadership, leader–member exchange (LMX), and psychological safety cannot be neglected as coping mechanisms to sustain the employee voice for mutual gains. To investigate ethical leadership’s mediating mechanisms and boundary conditions on voice behavior, we examined a moderated mediation model with the leader–member exchange as a moderator and psychological safety as a mediator. Grounded in social exchange theory (SET), the current study uniquely posits and tests that employees feel psychologically safe in the presence of an ethical leader with whom they have high-quality social exchanges. Data were collected from 281 employees from the public corporations and private enterprises of the petroleum sector of Karachi. Results of the analysis, through SPSS and AMOS, revealed that psychological safety mediated the relationship of ethical leadership and voice behavior, while the indirect effect of ethical leadership on voice behavior (via psychological safety) is stronger for those employees who enjoy high-quality exchanges with ethical leaders. LMX was also found to moderate the relationship between ethical leadership and voice behavior. Contributions, recommendations, and limitations of the current study and further research areas are also discussed. The study offers practical insight on the mechanism of ethical leadership on employee voice behavior and recommends leaders to develop social exchanges to improve voice behavior for sustainable success.

## 1. Introduction

An organization cannot indefinitely avoid changes under the current dynamic and highly competitive business conditions, and therefore creating and employing new ideas is crucial. Leaders should facilitate the prerequisites to change the status quo, welcome new ideas and help implement those ideas. Developing countries usually practice a bureaucratic style of leadership, in which case management is often disguised as leadership—and even more so in a country such as Pakistan, where high uncertainty avoidance, collectivism, and power distance norms prevail [1]. Research has established that developed countries score lower on the Power Distance Index (PDI) [2] but in developing nations like Pakistan, its influence has been established to be high [3,4]. This dimension is a reflection of an acceptance of hierarchical order in society and in workplaces [4]. According to researchers, in countries scoring high on the PDI such as Pakistan, employees are afraid and reluctant to show any disagreement with their managers or to raise their voice about concerns [3]. “The extent to which the members of a culture feel threatened by ambiguous or unknown situations and have created beliefs and institutions that try to avoid these” is measured by uncertainty avoidance [4]. Pakistan’s score on this dimension is 70 [5], which shows how threatened the employees here feel about raising their voice when faced with uncertain situations and ethical issues. Being a collectivist society, “where the society maintains a higher degree of interdependence among its members” [5], employees here score relatively low on creative self-efficacy, which hinders their ability to use their voice creatively [6]. A high-tech industry such as oil and gas requires constant innovations and up-gradations, whether it is located in Pakistan or in a developed country. Innovations and an environment encouraging voicing opinions regarding innovations are duly tied to a positive, ethical kind of leadership so that people can propose new ideas more freely. In this context, employees should be respected and treated fairly [7], so that they respond positively to their leaders [8]. Similar to the majority of the other developing countries, a workplace culture of being silent prevails in Pakistan and exercising one’s voice is not typically viewed as normal, mainly because of the costs and lack of leadership support.

Leaders are responsible for institutionalizing ethical standards and behaviors [9]; they play a critical role in shaping and maintaining an ethical culture in the organization [10]. Ethical leadership (EL) facilitates employees’ engagement and encourages them to speak up [11]. Many studies have demonstrated the impact of EL on-employee voice, for example, see [12,13]. Such leaders also welcome and acknowledge followers’ ideas, connect followers in decisions, delegate powers, and establish a principled and fair structure [14]. EL has significant contributions in promoting voice behavior, leading to the success or failure of organizational functions [15,16].

Ethical leaders maintain a safe climate for creating the necessary environment for voice behavior (VB). Psychological safety (PS) is a precondition that ethical leaders need to build in the organizational climate to encourage employees to propose new changes and ideas [17]. Creating and sustaining a psychologically sheltered environment and a sense of mutual respect is one of the core traits of EL. Such environments offer the followers a sense of being safe to speak up differently [18]. Safety against rebukes from coworkers or supervisors is vital to attract diverse views, opinions, or voices on any organizational matter. VB is subject to risks for both leaders and followers, so leaders have the ethical responsibility to provide a safe climate and encourage the followers’ voice. If leaders want their personnel to speak up, there must not exist any feelings of insecurity among employees [10].

Moreover, leader–member exchange (LMX) can affect the relationships between EL and VB. Graen and Scandura [19] have referred to LMX as exchanges between leaders and followers. Walumbwa et al. [20] consider LMX as an indicator of the effectiveness of social exchange relationships between leaders and their subordinates. LMX theory, as shaped by the relationships between leaders and subordinates, states that a leader establishes different types of relationships with diverse followers to yield diverse outcomes for the employees and their organizations. Based on the norm of reciprocity in social exchange relationships, an employee with a high level of LMX is more likely to repay his/her organization in the form of positive attitudes and constructive work behaviors [21] such as exercising one’s voice. Previous studies have taken EL as a precondition for LMX, for example, [22] and others. However, we propose and test LMX as a boundary condition on EL–VB and on EL–PS–VB, considering LMX as a theory distinct from ethical leadership.

VB is yet in its infancy in the oil and gas sector [23]. Afkhami Ardakani and Mehrabanfar [24] reported the prevalence of organizational silence in the Iranian petroleum industry due to bureaucratic obstacles. Fast et al. [25] found a positive relationship between managerial voice solicitations and employee VB in a USA petroleum firm. Mordi and Oruh [26] identified different themes of voice in Nigeria’s petroleum industry from both managerial and employees’ perspectives. According to their study, paternalism (accepting and reinforcing unequal power distribution between supervisors and employees) and high power-distance culture both influence voice behavior. Further, both these factors are said to impact the strategies facilitating employee voice [26]. In this context, there is a need to explore VB from the perspective of other developing countries, such as Pakistan, in which case VB is an under-researched concept. Pakistan, having a high power-distance cultural orientation, overwhelmingly practices a bureaucratic leadership style, so this study has contextual significance. Since the relationship of EL and VB has been studied through a piecemeal approach with PS and LMX, respectively, we have therefore proposed a combined moderated-mediation model which, to our knowledge, has not been studied yet. Against this backdrop, this study hopes to contribute to the existing literature on employee voice by linking the perceptions on interpersonal dynamics of how these safety feelings are constructed, understood, and facilitated by employees, for cordial employer–employee relationships.

By doing so, we add to the recent literature on ethical leadership and voice behavior in seven ways: (i) We bring literature together with related theories by examining ethical leadership and psychological safety as determinants of voice behavior. (ii) We extend the model by integrating ethical leadership and LMX (as contextual inputs/situational influences) and psychological safety (as a process or individual perception) that might be stimulated by the interaction of ethical leadership and LMX. The rationale behind this is that the processes (i.e., mediation) through which ethical leadership has been documented to exert its impact have been explored independently from the boundary conditions (i.e., moderators) under whose influence these processes may operate. Since the contextual determinants influence the effectiveness of leadership and its processes, a combined (moderated-mediated) process will help in furthering our understanding of ethical leadership. (iii) We include psychological safety as a possible mediator between ethical leadership and voice behavior which happens to be the final outcome. (iv) We embrace LMX as a possible moderator of ethical leadership and voice behavior. (v) We move beyond the group-oriented approach on voice literature in the recent past [27]. Existing studies on EL and VB have taken up a group-focused prospect and have investigated voice as a “shared unit property”, for example, in [27,28], but voice inherently is supposed to belong to self-initiated actions [29]. Thus, examining voice processes from a shared perspective undermines individual motivation and presumes that the homogeneous processes operate as far as the driving forces and manifestations of voice behavior are concerned [28]. Additionally, analyzing EL–VB at the group level assumes the uniformity of influencing mechanisms exercised by leaders and followers’ reactions to them, ignoring the significance of the interpersonal nature of leaders’ relationships with their followers [30]. This study argues and tests individualized influences of ethical leadership on employees’ voice behavior via employees’ sense of psychological safety and under the influence of one-to-one LMX interactions. (vi) We test the model in the oil and gas sector (a relatively unexplored industry of Pakistan regarding voice behavior). (vii) Expansion of the ethical leadership–employee voice research in a different context (e.g., in the country of Pakistan and culture which is collectivist and power distant) is the final contribution. Notably, voice behavior-related research conducted in Pakistan is insufficient compared to that conducted in other developed countries of the globe. Hence, now there is a need to expand the research context which will enhance the explanatory potential of ethical leadership, LMX, and psychological safety in promoting voice behavior and applicability of our theoretical framework in the oil and gas sector of Pakistan.

## 2. Literature Review

Social exchange theory (SET) [31] states that trusting or transactional relationships are developed among members of the organization based on mutual experiences and norms of reciprocity [32]. Those relations could be financial benefits and/or social networks [33]. SET also confirms the idea of followers copying and internalizing the behavior they observe in their leaders. Followers reciprocate more when they are treated carefully and fairly.

EL is rooted in and aligned with SET in that EL behavior drives the ethically sound behavior of the employees. Therefore, leaders can support the values and norms of the organization and can even change the organizational culture. SET outcomes also include VB [34,35,36]. EL is positively linked with VB [37]. PS is also rooted in SET [38], and the entirety of the social exchange system influences the employees’ PS [39]. EL is the predecessor to PS [18]. An individual’s psychological perception of the organizational climate, whether safe or not, has an impact on choosing VB as a social exchange [40]. PS has been part (a mediator) of a wider social exchange process, including EL and VB [18]. LMX characterizes the strengths of exchange relationships between employees and their supervisors [19]. LMX is the extent of the social exchange relationships between supervisors and subordinates with the prospect to impact subordinates’ conduct and sense of obligation [41]. Leaders’ relationships with employees are nurtured and developed over time varying from employee to employee, and can broadly be seen as either high-quality or low-quality social exchange relationships.

Theoretically, there are two main reasons for leadership behavior affecting the followers’ VB [42]. Firstly, speaking up comprises sharing ideas and thoughts with superiors/leaders for assumed allocation of resources to the identified concerns [43]. Secondly, leaders have control over followers’ salaries, appraisals, duties, and promotions, which signals to the followers that their voices can bring reprimands or rewards administered by their leaders [44]. VB is a central tenet of EL [45]. Ethical leaders provide a voice to their followers [30]. They express high ethical standards, encouraging the employees to express their views on the existing situations and propose new ideas of improvement on ethical matters, work contexts, and processes. Brown et al. [30] described that ethical leader have an important relationship with employees’ readiness to report workplace problems to their management, which is a part of the VB concept [45]. Empirically, EL has a positive impact on VB (e.g., [11,37,46,47,48,49,50,51]). So, we can hypothesize that EL increases the level of employees’ voice behavior in the workplace:

**Hypothesis** **1.** *Ethical leadership significantly predicts voice behavior*.

Leaders who promote employee inclusiveness increase the sense of psychological safety by diminishing the effects of status [52]. Inclusiveness also elevates decisions’ quality and favors learning from failures [53]. An employee feels safe and productive when able to express his/her view or voice, and s/he does gain psychological benefits in the process. A relationship emerges as ethical leaders promote a climate of taking responsibility for one’s work assignments, clarify behavioral roles and accepted norms, and articulate moral standards [8]. Such clarity reduces uncertainty and cultivates a psychologically safe climate [54]. Ethical leaders improve mutual trust, communicate with openness, respect their followers, show genuine concern for them, consider their personal situations, and provide emotional and instrumental support to the followers. Thus, they promote a psychologically safe climate by adopting these behaviors [30,38]. Through leaders’ enactment of these behaviors, followers feel respected and valued, thereby developing a shared perception of PS, leading to the expression of their true selves [55]. Empirical evidence supports these arguments, e.g., [18,56,57], hence, we hypothesize:

**Hypothesis** **2.** *Ethical leadership significantly predicts psychological safety*.

Edmondson [34], referred to psychological safety as the employee’s faith that his voice will not be disregarded by his colleagues, supervisor, or any other member of his team. Such a voice could be an inquiry, feedback, reporting a discrepancy, or proposing a new and positive idea [58,59,60]. Hence, employees will be more involved in voice behavior when they sense that the negative implications associated with speaking up are minimal, in which case they will find it more convenient to express their points of view, whereas they would prefer silence when they feel the opposite [34,60,61]. Employees count the costs and benefits before they speak, and thus psychological safety is described as a vital factor that can influence the employees’ voice [62]. For example, employees opt for defensive silence instead of speaking up if they fear important personal losses such as restricted career growth and loss of social facilitation from colleagues and superiors. Leaders’ gestures or behaviors are the indicators which employees use to examine if volunteer expression of the unsolicited information is safe or unsafe, as usually the power holders have the compensating and approving authority [15]. Leaders who are keen to involve their followers, personally acknowledge their inputs, carefully notice their efforts, and reciprocate with appropriate actions indicating that speaking the truth is not always harmful or risky [58,63]. Such collaboration minimizes the risks even greater in high power-distance cultures and enhances VB [64,65]. Many scholars have empirically validated the PS and VB relationship (e.g., [17,18,48,66,67,68,69,70,71]). To further validate the hypothesis, we developed the following:

**Hypothesis** **3.** *Psychological safety significantly predicts voice behavior*.

Voice behavior is a deliberate act that takes into account its implications, i.e., what can organizational members win or lose by raising their voice over a certain matter. Detert and Burris [59] stated that psychological safety would be understood as a belief which safeguards risky behaviors such as raising one’s voice against the potential harms to the participating individuals. Edmondson [34] further elaborated this belief as a “shared belief that the team is safe for interpersonal risk-taking.” To create a psychologically safe atmosphere, the leader plays a vital role by elevating psychological trust through the removal of obstructions that can thwart the expression of followers’ ideas. Kark and Carmeli [71], described that feeling psychologically safe helps the employees to manage their stress and utilize new ideas and suggestions in a better way. Walumbwa and Schaubroeck [18] stated that feeling psychologically safe is an environment that mirrors high-level trust and mutual respect at the workplace.

Consequently, the factor of psychological safety mediates the relationship between a leader’s behavior (deemed as external stimulus) and a follower’s choice of staying silent or speaking up (an internal stimulus). Such perception confirms the findings of Podsakoff et al. [72], who described that followers’ faith in leadership—where faith is taken as a factor equivalent to psychological safety—assures others that leaders will not harm followers upon voicing their views or similar actions. According to multiple studies, psychological safety mediates the relationships between ethical leaders and voice behavior [17,18,73], but it should be noted that this finding is not unequivocal and was not replicated in the most recent papers on this topic [48]. Taking into account the abovementioned results, we postulated that:

**Hypothesis** **4.** *Psychological safety mediates the relationship between ethical leadership and voice behavior*.

LMX can influence followers’ behavior and commitments through healthy interactions and gauges the extent and effectiveness of social exchange relationships between leaders and their followers [19,41]. According to LMX theory, there exist disparities in social exchange relationships when leaders and followers interact [74,75]. Owing to these disparities, the leader–member social exchange relationships can be described either as high-quality exchanges or low-quality exchanges. The ethical leader will be more likely to enable followers to define themselves in terms of the leader–follower relationship. Followers in high-quality exchange relationships with their leaders experience the leaders’ concern, liking, and care, which proves beneficial in developing followers’ confidence in their own capabilities. Such trust, care, and concern from the leaders in high-quality exchanges persuade followers to imitate leaders’ actions [76]. Not only is greater autonomy experienced by these followers [77], but they are the recipients of enhanced and useful developmental feedback from the leaders, which additionally causes an increase in followers’ self-efficacy [78], and hence an increase in exercising voice behavior. On the contrary, followers in low-quality exchange relationships with their leaders experience less effective interactions, are not frequently guided, feel less supported by their leaders, and are assigned fewer responsibilities on account of distrust [79], all of which reduces their opportunities to exercise their voice.

The above statement proposed by LMX theory can be extended to ethical leaders and to those group members who would perceive ethical leaders as trustworthy and attractive, begetting effective and greater interaction, and benefiting highly from ethical leaders’ conduct and hence receiving more opportunities to speak up. On the other hand, some will benefit less from their ethical leaders, namely those in low-quality relationships, which would limit their willingness to speak up. Moreover, observing at the scale level, the items of these two constructs, ethical leadership (e.g., “My leader makes fair and balanced decisions”) and LMX (e.g., “My supervisor and I are suited to each other”), happen to be independent and different from each other. Ethical leadership accounts for a leader’s overall moral conduct, whereas LMX demonstrates a leader’s relationship quality with a particular follower. This study, therefore, assumes that an interaction exists between LMX and ethical leadership. Hence, LMX, by affecting followers’ receptiveness to the influence of ethical leaders, is hypothesized to moderate the relationship between ethical leadership and followers’ voice behavior. In this situation, the relationship is expected to be stronger for the employees having high-quality social exchanges with their leaders. The existence of and implications for differences in the quality of relationships between ethical leaders and their followers have yet to be fully explored [80]. Additionally, using the socio-contextual lens, high-quality LMX can be viewed as a contributing factor to strengthen the impact of ethical leadership on followers’ VB. A low-quality LMX exchange, conversely, serves as an inhibitor that weakens the relationship of EL and employees’ VB.

Previous empirical research revealed that employees would engage in VB when they sensed high-quality LMX relationships with their supervisors [81]. In contrast to this, subordinates having low-level LMX relationships with their supervisors usually hesitate to use their voice [74]. Instead of using LMX as a moderator, the extant literature, with the exception of Neubert et al. [82], has rather used LMX as a mediator (e.g., [51,83]) in the EL–VB relationship. Nazir et al. [84], while studying benevolent leadership and VB, suggested using LMX as a moderator with other positive types of leadership. In response to these, we have developed the following:

**Hypothesis** **5.** *LMX will moderate the relationship of ethical leadership with voice behavior*.

Ethical leaders care about the psychological well-being of their followers [10]. LMX, as the crucial interacting unit, acts as a vehicle for both the conception and further development of psychological safety perceptions among the employees. Through LMX, ethical leaders become able to exert positive psychological influence over employees [85]. Since high-quality LMX exchanges build supportive and trusting relationships, employees with whom ethical leaders enjoy high-quality LMX exchanges feel psychologically safe. These employees enjoy more access to relevant information, as ethical leaders give them the right conditions to work. These right conditions range from the provision of flexible work arrangements to the authority to take new initiatives with the acceptability of even failing at them without fearing embarrassment, retaliation, and negative repercussions [54], all of which contribute to psychological safety perceptions. Thus, we contend that such employees with the right information are better positioned to give relevant work ideas or even question wrong work processes. Hence, Uhl-Bien and Maslyn [86] argue that the natural outcome of psychological safety is to drive voice behavior. While this may be easier said than done, we assert that employees who perceive greater psychological safety influenced by LMX with ethical leaders can arguably be better equipped and confident to raise their voice.

Previous literature has validated the relationship between LMX and psychological safety [87]. PS has also been studied as a mediator in the relationship between LMX and VB e.g., [88,89]. Yet, to the best of our knowledge, only Neubert et al. [82] have reported the moderation of LMX on the relationship of EL with VB through a mediator, i.e., a promotion focus (moderated-mediation model). Another empirical study, by Niu et al. [90], found LMX to be moderating the mediated relationship between inclusive leadership and VB. Given that LMX is influential in facilitating psychological safety coupled with the non-existence of empirical validation of our proposed model, we confidently propose that:

**Hypothesis** **6.** *LMX will moderate the relationship between ethical leadership and voice behavior mediated by psychological safety, such that voice behavior will be high with the high values of LMX and vice versa*.

## 3. Materials and Methods

Petroleum exploration and production activities date back to the inception of Pakistan and the oil and gas sector is among the biggest sectors of Pakistan’s economy. The total energy supply during 2019 was about 86 million tons of oil equivalent. To understand the impact of gas’s contribution, indigenous gas is about 35% of the total energy supply. The local exploration and production (E&P) industry produces about 4 billion cubic feet (BCF) per day of gas. The refineries’ total requirement for crude oil is about 400,000 barrels, of which the local E&P industry supplies about 20%. The local E&P industry also produces about 75% of LPG demand. It can thus be concluded that the oil and gas industry is the backbone of the country in every respect, including in its contribution to revenues and taxes, as all E&P companies operating in Pakistan are among the top taxpayers of the country [91]. Considering the high significance of this sector in the economy and growth of the country, the study of the voice behavior—a lack of which could be fatal for the industry and the country at the same time—is also significant and relevant. Although petroleum activities are carried out across Pakistan, companies’ corporate offices are located in the major cities of Karachi, Islamabad, and Lahore, etc. We chose the Karachi petroleum industry due to better data availability regarding national and international companies. We formally requested the willingness of the respondents to participate in a survey to provide the primary dataset, along with a brief on the study’s motives of psychological realism which could contribute to the external validity of the research. Respondents were sent email reminders to mitigate the nonresponse bias. There were 30 questions in this survey, so we targeted a response of around 300, according to a common recommendation of 10 responses for each survey question for a suitable sample size. We received 281 responses from the 300 distributed questionnaires from the middle management employees chosen from six oil and gas sector companies. The response rate (i.e., >70%) [92] further reduced the nonresponse bias from randomly selected managers. Respondents were composed of 83% male and 17% females, and most belonged to the age groups 41–50 and 31–40, with 47% and 39% representation, respectively. More than 52% of the respondents had 11–15 years of experience. The sample size was calculated through Yamane’s (1967) formula (i.e., *n* = N/1 + N × (e)^2^; [93] where *n* is the sample size, i.e., 300 approx., N is the population, i.e., 1200 middle management employees approx.), under the simple random sampling technique which is used for an unbiased representation of the population group, and also helps in improving the external validity. The list of employees was randomized through MS Excel; every 4th random person from the list was chosen as a respondent.

For each measure, respondents were assessed on their agreement with various statements, for each set on a five-point Likert-type scale, with a response format ranging from “1 = strongly disagree” to “5 = strongly agree” (see Appendix A Table A1). Ethical leadership was assessed by using Brown et al.’s [30] 10-item scale (see Appendix A Table A2), and its reliability was 0.81. A sample item is, “My leader disciplines employees who violate ethical standards.” Voice behavior was measured with Van Dyne and LePine’s [45] 6-item scale (see Appendix A Table A3). A sample item is, “I speak up and encourage others in this group to get involved in issues that affect the group.” We found Cronbach’s value was 0.81. Psychological safety was measured by using a 7-item scale developed by Edmondson [34] known as the Psychological Safety Scale (PSS) (see Appendix A Table A4). A sample item is, “If you make a mistake, it is often held against you.” We found its Cronbach value as 0.82. LMX was measured by using the 7-item LXM scale developed by Scandura and Graen [94] (see Appendix A Table A5). A sample item is, “How well do you feel that your immediate supervisor understands your problems and needs?” We found Cronbach’s alpha had a value of 0.82.

## 4. Results

Before proceeding to the analysis, we fulfilled the model assumptions, e.g., normality (i.e., skewness with maximum z-score of 2.21 at *p* < 0.05 for any variable, and kurtosis with a maximum z-score of 2.79 at *p* < 0.01 for any variable. These significance levels are defined by Field and Miles [95]). Non-collinearity (i.e., tolerance with a minimum value of 0.84 > 0.2 threshold set by Menard [96] for any predictor), and VIF (with a maximum score of 1.19 < 3.3, a threshold value set by Kock [97] for any predictor) assumptions were also met. Linearity and homoscedasticity were fulfilled as well. As our data came from one common source in a self-rated mode, it was inclined to have priming effects, so evaluation apprehension and socially desirable responses might have contributed to common method variance (CMV) [98]. To address this issue, we pooled all the items of the four constructs into a single factor for factor analysis. The outcomes of the one-factor model displayed a poor model fit (χ2 (1391)/df (375) = 3.71, CFI = 0.83, TLI = 0.79, RMR = 0.053, RMSEA = 0.098). The cumulative variance was 47%, while maximum variance by any single factor was 20% i.e., <50% [99], confirming that data was free of CMV.

Then we conducted the confirmatory factor analysis (CFA) with AMOS v. 24. Table 1 shows that the four-factor model had χ^2^ = 684 and df = 367, yielding 1.86 against the criteria value of <5; it had CFI = 0.95 and TLI = 0.93 against the acceptable value of 0.90; RMSEA = 0.056 and SRMR = 0.034 were duly within the acceptable range, i.e., <0.07 and <0.05 respectively [100]. The four-factor model best fitted the data versus alternate models (see Table 1); hence, our measures’ discriminant validity was supported. Composite reliability was also above the baseline of 0.6 [101], which indicated that all scales were internally consistent. Means, standard deviations, correlations among study variables, and composite reliability are reported in Table 2.

### Hypothesis Testing

The results of preliminary analyses suggested that all study variables were distinct but correlated with each other, so we proceeded with the testing of hypotheses. We utilized the structural equation modeling (SEM) approach in AMOS v.24 to test our hypotheses and used centered variables [102] for more meaningful analyses [103]. Separate analyses of mediation and moderation models could have issues with the fitness of the overall model when the paths are estimated simultaneously [104,105]. Therefore, we used Edwards and Lambert’s [105] “direct effect and first stage moderation model” to combine the moderation and mediation for more substantive answers to the theoretical queries (p. 4). As suggested by SEM, a simultaneous test of the significance of the path from an IV to a mediator and the path from the mediator to DV relatively provides the best balance of Type I error rates and more statistical power [106].

**Figure 1:**
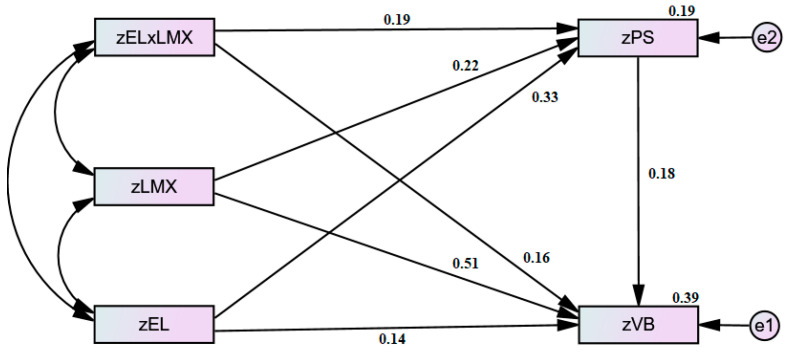
Moderated mediation.

Employee demographic variables (e.g., education, gender, age) could influence voice behavior [59,107] so we controlled gender and education for our study, which were found to be related to VB, and suggest that future studies should also use such controls to avoid omitted variable bias. Hayduk [108] suggested testing and comparing other alternative models in SEM, so we estimated the plausibility of four different alternative models. Table 3 has the statistics for all models, and our proposed moderated-mediated model was the best fit for further interpretation (see Figure 1).

Statistics for Hypotheses 1–3 are given in Table 4 and Table 5, which show that all paths (superscript a, b, and c) were significant and critical ratio values (CR) for all paths were also greater than 1.96, which approved all of the direct hypotheses. Results for Hypothesis 4 on PS mediation between EL and VB can be noted from Table 4. The CR value of total effect was 3.33 (>1.96), indicating that mediating effect was significant; likewise, CR value 2.4 (>1.96) of indirect effect was also significant. CR value of direct effects was 2.12 (>1.96), whereas values at lower and upper bounds of both bias-corrected and percentile bootstraps’ confidence intervals (CIs) contained no zero, their *p*-values were also <0.05, so we concluded it as a partial mediation [109]. Sobel’s (1982) test further confirmed the significant indirect effect of EL on VB via PS (z = 3.01, *p* < 0.003), supporting Hypothesis 4.

**Figure 2:**
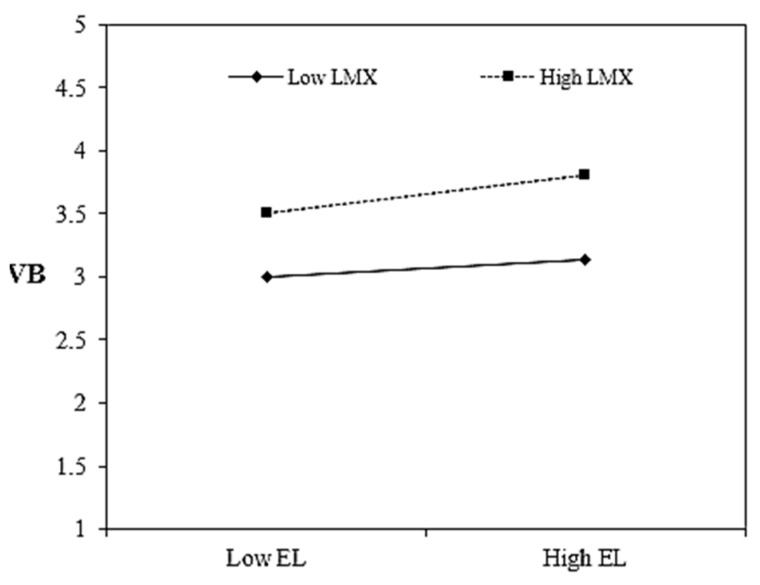
Direct effect interaction. Note: EL = ethical leadership, LMX = leader–member exchange, PS = psychological safety, VB = voice behavior.

Hypothesis 5 anticipated that LMX would moderate the relationship of EL and VB (direct effect moderation); Table 5 shows that the interaction effect (i.e., EL × LMX) met the condition of significance [110] and positively affected the VB (b = 0.16, *p* = 0.034). We used bias-corrected bootstrap with 95% CIs to examine the conditional effect of EL on VB at the different levels of LMX. As given in Table 5, the impact of EL on VB was insignificant (b = −0.04, *p* = 0.606) at the lower levels of LMX; the same impact was increased and significant (b = 0.28, *p* = 0.014) at higher levels of LMX. We plotted the interaction effect to further evaluate our hypothesis [111]. Figure 2 provided evidence that the impact of EL on VB increased as LMX increased. Slope gradient was 0.14 (t = 2.56, *p* = 0.011) at −1 SD of LMX, whereas the same was 0.30 (t = 3.85, *p* = 0.000) at +1 SD of LMX, which candidly confirmed that EL’s impact was stronger at the higher levels of LMX. These analyses together provided support to Hypothesis 5.

**Figure 3:**
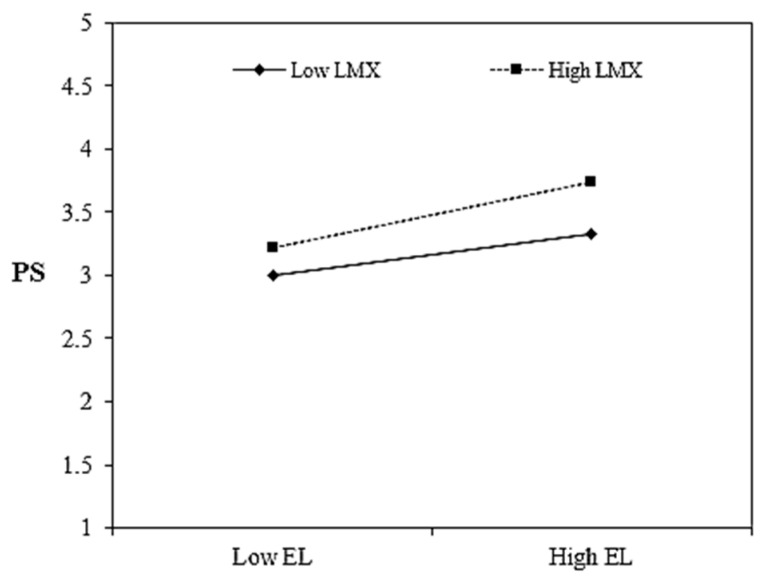
1st stage interaction. Note: EL = ethical leadership, LMX = leader–member exchange, PS = psychological safety, VB = voice behavior.

Hypothesis 6 was about the moderated role of LMX on a relationship (mediated by PS) between EL and VB. Interaction effect (EL × LMX) was positive and significant (b = 0.19, *p* = 0.012), confirming that 1st stage moderation was successful (Table 5). The conditional effect of EL on PS was not significant (b = 0.12, *p* = 0.310) when LMX was low, but it was increased and significant (b = 0.50, *p* = 0.001) when LMX was high, indicating that PS perception increases with the increases in LMX. We plotted (Figure 3) this interaction effect and results confirmed that the impact of EL on PS was stronger (slope = 0.52, t = 6.67, *p* = <0.001) at +1 SD of LMX as compared with −1 SD of LMX (slope = 0.33, t = 6.03, *p* = <0.001). Preceding results enabled us to analyze the remaining parts of our model. We utilized bias-corrected (BC) and percentile method (PM) bootstrap CIs to check the conditional indirect effect (CIE) of EL on VB via PS at different levels of LMX. As shown in Table 5, CIE was not significant (BC: b = 0.02, *p* = 0.233; PM: b = 0.02, *p* = 0.282) when LMX was low; in contrast to this, CIE was not only significant but also stronger (BC: b = 0.09, *p* = 0.001; PM: b = 0.09, *p* = 0.001) when LMX was high. We further examined the index of moderated mediation [103] which was also significant for both BC and PM bootstrapping. Taken together, these results confirmed the approval of Hypothesis 6. For more details, please see Appendix A, Table A1 and Table A5.

## 5. Discussion

The rising support in favor of ethical leadership as a promising leadership style to induce positive behaviors among employees is undeniable [8,20]. However, research explaining the underlying psychological processes of ethical leaders’ positive influence is still insufficient [20], particularly regarding ethical leadership’s influence on VB. Additionally, ethically oriented behavior by leaders may not be constant across different situations, times, and personalities while exchanging interactions with different followers. Hence, the intensity and frequency of psychological safety perceptions will vary while interacting with ethical leaders depending upon the quality of leader–member exchanges. Among the different kinds of voice behavior patterns examined in the literature, psychological safety can be considered fundamental for understanding the specific nature of VB. Our study makes three important contributions to the literature on EL and VB. By investigating the role of LMX, this study significantly expands the knowledge about how ethical supervisors can cultivate perceptions of psychological safety for exercising voice. With the exception of some studies by Walumbwa and Schaubroeck [18], Jian [73] and Sağnak [17], relatively little attention has been paid to how psychological safety mediates the influence of ethical leadership on VB. The current investigation makes important assertions in this direction and complements the existing literature. Secondly, interactions with leaders at the workplace appear to provide a ready and safe interpretation of events regarding when employees raise their voice. High-quality LMX becomes an immediate reference to measure the approachability of the supervisor–subordinate dynamic and dyadic context. This way we extend the work of Neubert et al. [82] on moderation of LMX on EL-promotion-focused VB by explicating the interaction between LMX and EL in predicting VB through the mediation of psychological safety. These have implications in terms of followers’ attitudes about upward communication evaluation and impact the members’ propensity to raise their voices about relevant matters with the top management. Further, our study extends past research on EL and VB by establishing LMX as a moderator between these two. Under high-quality LMX exchanges, ethical leaders exercise strong influence on VB; however, the impact becomes insignificant under poorer LMX relationship with ethical leaders. This finding is again in line with the study of Neubert et al. [82] on moderation of LMX and EL for predicting regulatory mindsets. However, our study is novel for exploring this interaction for VB. By demonstrating that LMX moderates the relationship of EL to VB and of EL–PS to VB, we extend the theory to include situational factors and affirm a commonsense maxim that leadership influence is stronger in the context of quality relationships.

### 5.1. Theoretical Implications

This research contributes to the leadership literature by focusing on EL and demonstrating its role in determining followers’ voice behavior. The finding that ethical leadership can encourage subordinates’ voice behavior provides authentication that leadership ethics play a considerable part in establishing employee engagement in voice behavior. Similarly, ethical leadership can encourage employees’ voice behavior by enhancing their psychological safety. The current research highlights the moderating role of LMX in the affiliation of ethical leadership; as such moderation elevates the perceptions of PS and signifies that the higher the levels of LMX, the higher the chances of voice behavior. In short, this study confirmed that EL, LMX, and PS are correlated and have precursory properties for VB, whether these are studied in piecemeal or in combined models such as in ours, and to the best of our knowledge, this research has been among the pioneers testing the proposed model.

### 5.2. Practical Implications

From a practical perspective, this research provides insight into the management of knowledge workers. The growing importance of voice behavior for efficient operation and survival of organizations has increased the significance of knowledge workers around the world. Investigating factors that encourage such desired behavior in high-tech organizations is crucial. Thus, the current research has both theoretical and empirical evidence on the role of ethical leadership in positively and significantly affecting employees’ voice behavior. This research advocates the hiring, development, and promotion of ethical leaders/practices, along with sustaining environments of psychological safety and positive leader–member exchanges. This in turn can induce an overall ethical climate that will embed trickle-down effects across organizational hierarchies [112]. Individual employees will thus recognize organizational policies for establishing extra-role efforts to express favorable behaviors such as voicing opinions for overall organizational improvements.

### 5.3. Limitations and Future Prospects

There are some limitations of the present research. First, this study uses a cross-sectional design which indicates that causal inferences should be interpreted with caution; hence a longitudinal/time-lag design or mixed-method study can be designed in the future. Second, data for study variables were collected in a self-rated mode from the same source and from individuals in subordinate roles, which may raise concerns about common method bias/variance. Although the single factor test found no major concern about CMV, researchers may opt for triangulation techniques (e.g., data and methods, etc.) for better results in future studies. Third, because our paper is potentially the first study on the proposed model, generalization of the results is limited, so replication in other sectors is recommended. Fourth, as the results suggested, LMX was more likely to instigate voice behavior, but it is not certain if in-group LMX and out-group-LMX had the same impact on employees’ voice. Fifth, the moderating role of LMX in ethical leadership–voice behavior relationships might be stronger in Pakistan due to high power distance and collectivist cultural orientations, and subsequently, cross-cultural validation and generalization are therefore advised. Sixth, PS mediation between EL and VB was partial, and finding such partial mediation suggests that other self-regulatory processes and additional mechanisms may play a part in explaining the relation of these variables fully, but studying them was beyond the scope of our study. So, to supplement our findings, the effects of EL on the variables of this study and on other psychological processes in intervening roles or as boundary conditions should be explored in further research. Seventh, an important issue is that employees are not randomly assigned into workplaces. Failure to account for sorting of employees could bias estimated effects for the measures of well-being at work [113] as it may have biased the voice behavior in our case. Yet, as in many other studies, we investigated and explored the general phenomenon in the present research. Nonetheless, for future research, a more thorough research design is recommended. Further, the results may suffer from omitted variable bias; hence, future studies should use additional controls such as personality dimensions while testing the model. Lastly, we suggest replicating this research using other proximal VB antecedents, such as perceived organizational support [114]. Moreover, EL had a more direct influence on PS as compared with VB, so we leave another question for future research, i.e., “whether EL is more robust in predicting VB or PS”. It is worth mentioning that Avey et al. [10] indicated a similar finding on psychological ownership.

## 6. Conclusions

Despite the growth in EL research, more research is still needed due to the importance of EL for organizations [80]. Ethical behavior plays an important role in determining subordinates’ VB, as investigated in the current research, and findings can have significant implications for knowledge-intensive organizations such as the oil and gas sector when individuals speak their minds. By demonstrating PS as a significant mediator, our research identifies a proximal antecedent of voice behavior that can be enhanced through EL practices. Practicing EL paves the path for enhancing subordinates’ perceived empowerment which can instill a ‘can do’ perspective among followers for extra-role efforts such as raising their voice. The moderating role of LMX suggests that managers and leaders are required to focus on the nature of the relationship they have with subordinates to encourage followers’ VB. Research findings concluded that EL encourages followers to participate in VB. The positive relationship between EL and VB can also be attributed to distinguished leadership and psychological characteristics (e.g., LMX and PS), which provide followers with the cues regarding safety and effectiveness of voice.

## Figures and Tables

**Table 1:** CFA models comparison.

Model	χ^2^/df	Δχ^2^	TLI	CFI	RMR	RMSEA
4-Factor: EL, LMX, PS&VB	684/367 = 1.86	-	0.93	0.95	0.034	0.056
3-Factor: EL + LMX,PS &VB	1090/366 = 3.0	406	0.85	0.88	0.071	0.084
2-Factor: EL + LMX + PS&VB	1171/366 = 3.2	487	0.83	0.87	0.074	0.089
1-Factor: EL + LMX + PS + VB	1391/375 = 3.71	707	0.79	0.83	0.053	0.098

**Δ** variation among models. Note: EL = ethical leadership, LMX = leader–member exchange, PS = psychological safety, VB = voice behavior.

**Table 2:** Means, standard deviations, correlations, and composite reliability.

Variable	Mean	SD	1	2	3	4
EL	3.56	0.43	(0.91)			
LMX	3.10	0.41	0.14 **	(0.87) *		
PS	4.05	0.37	0.36 *	0.23 *	(0.80)	
VB	4.16	0.49	0.27 *	0.54 *	0.37	(0.94)

*n* = 281, * *p* < 0.001, ** *p* < 0.05, composite reliability is in parentheses. Note: EL = ethical leadership, LMX = leader–member exchange, PS = psychological safety, VB = voice behavior.

**Table 3:** SEM model comparison.

Model	χ^2^/df	Δχ^2^/Δdf	GFI	CFI	RMR	RMSEA
Mediation EL–PS–VB	116/8 = 14.5	112/4	0.91	0.51	0.11	0.22
Direct Effect Moderation EL–LMX–VB	77/8 = 9.6	73/4	0.94	0.68	0.11	0.18
1st Stage Moderation EL–LMX–PS	142/8 = 17.7	138/4	0.88	0.39	0.14	0.24
Moderated-Mediation EL–LMX–PS–VB	4/4 = 1	-	0.99	1	0.02	0.00

Note: EL = ethical leadership, LMX = leader–member exchange, PS = psychological safety, VB = voice behavior.

**Table 4:** Mediation analysis.

EL-PS-VB	*B*	SE	CR	Bias-Corrected 95% CI			Percentile 95% CI		
				LLCI	ULCI	*p*	LLCI	ULCI	*p*
Total Effect	0.20	0.060	3.33	0.088	0.323	0.001	0.076	0.309	0.002
Direct Effect	0.14	0.066	2.12	0.016	0.277	0.028	0.001	0.260	0.048
Indirect Effect	0.06	0.025	2.40	0.021	0.119	0.001	0.021	0.118	0.001

Note: EL = ethical leadership, LMX = leader–member exchange, PS = psychological safety, VB = voice behavior.

**Table 5:** Moderated-mediation analyses.

Variable	Psychological Safety					Voice Behavior				
	B	SE	LLCI	ULCI	*p*	B	SE	LLCI	ULCI	*p*
Gender						−0.07	0.048	−0.165	0.018	0.131
Education						0.02	0.047	−0.071	0.115	0.615
EL	0.33 ^a^	0.054	0.211	0.455	0.001	0.14 ^c^	0.050	0.001	0.260	0.048
LMX	0.22	0.055	0.100	0.337	0.001	0.51	0.049	0.398	0.614	0.001
EL × LMX	0.19	0.056	0.047	0.336	0.012	0.16	0.049	0.010	0.298	0.034
PS						0.18 ^b^	0.052	0.070	0.303	0.001
	*R^2^* = 0.20					*R^2^* = 0.39				
LMX	Conditional Effect of EL on PS					Conditional Effect of EL on VB				
−1 SD	0.12	0.11	−0.101	0.341	0.310	−0.04	0.07	−0.179	0.101	0.606
Mean	0.31	0.07	0.175	0.433	0.002	0.12	0.06	0.006	0.249	0.045
+1 SD	0.50	0.08	0.353	0.675	0.001	0.28	0.12	0.056	0.502	0.014
	Conditional Indirect Effect of EL on VB via PS									
LMX	Bias-Corrected 95% CI					Percentile Method 95% CI				
−1 SD	0.02	0.03	−0.013	0.088	0.233	0.02	0.03	−0.015	0.083	0.282
Mean	0.06	0.02	0.019	0.114	0.001	0.06	0.02	0.018	0.114	0.001
+1 SD	0.09	0.03	0.034	0.165	0.001	0.09	0.03	0.034	0.164	0.001
	Index of Moderated Mediation									
	0.04	0.02	0.010	0.077	0.006	0.04	0.02	0.007	0.071	0.012

Note: EL = ethical leadership, LMX = leader–member exchange, PS = psychological safety, VB = voice behavior.

## Data Availability

The data will be made available on request from the corresponding author.

## Appendix A

The data will be made available on request from the corresponding author.

Kindly read the following statements carefully and respond by encircling the relevant score box which you feel most genuine and appropriate to your experience.

**Table A1:** Five-point likert scale.

1	2	3	4	5
Strongly Disagree (SD)	Disagree (D)	Neutral (N)	Agree (A)	Strongly Agree (SA)

**Table A2:** Ethical leadership scale.

Sr.	The Following Questions are Related to Your Leader/Supervisor/Manager, Who	SD	D	N	A	SA
1	Conducts his/her personal life in an ethical manner.	1	2	3	4	5
2	Can be trusted.	1	2	3	4	5
3	Asks what is the right thing to do? When making decisions.	1	2	3	4	5
4	Listens to what employees have to say.	1	2	3	4	5
5	Has the best interest of employees in mind.	1	2	3	4	5
6	Defines success not just by results but also the way that they are obtained.	1	2	3	4	5
7	Makes fair and balanced decisions.	1	2	3	4	5
8	Discusses business ethics or values with employees.	1	2	3	4	5
9	Sets an example of how to do the things the right way in terms of ethics.	1	2	3	4	5
10	Disciplines employees who violate ethical standards.	1	2	3	4	5

**Table A3:** Voice behavior scale.

Sr.	The Following Questions Concern Your Contributions at the Workplace	SD	D	N	A	SA
1	I develop and make recommendations to my supervisor concerning issues that affect my work.	1	2	3	4	5
2	I speak up and encourage others in my work unit to get involved in issues that affect our work.	1	2	3	4	5
3	I communicate my opinions about work issues to others in my work unit, even if their opinions are different and they disagree with me.	1	2	3	4	5
4	I keep well informed about issues at work where my opinion can be useful.	1	2	3	4	5
5	I get involved in issues that affect the quality of life in my work unit.	1	2	3	4	5
6	I speak up to my supervisor with ideas for new projects or changes in procedures at work.	1	2	3	4	5

**Table A4:** Psychological safety scale.

Sr.	The Following Questions are Related to How Much You Feel Psychologically Safe in the Workplace	SD	D	N	A	SA
1	If you make a mistake on this team, it is often held against you.	1	2	3	4	5
2	Members of this team are able to bring up problems and tough issues.	1	2	3	4	5
3	People on this team sometimes reject others for being different.	1	2	3	4	5
4	It is safe to take a risk on this team.	1	2	3	4	5
5	It is difficult to ask other members of this team for help.	1	2	3	4	5
6	No one on this team would deliberately act in a way that undermines my efforts.	1	2	3	4	5
7	Working with members of this team, my unique skills and talents are valued and utilized.	1	2	3	4	5

**Table A5:** LMX scale.

Sr.	The Following Questions Concern Relationships in the Workplace					
1a	Do you usually feel that you know where you stand?	Never Know	Seldom Know	Neutral	Usually Know	Always Know
		1	2	3	4	5
1b	Do you usually know how satisfied your immediate supervisor is with what you do?	Highly Unsatisfied	Unsatisfied	Neutral	Satisfied	Highly Satisfied
		1	2	3	4	5
2	How well do you feel that your immediate supervisor understands your problems and needs?	Never Understands	Sometime Understands	Neutral	Understands	Fully Understands
		1	2	3	4	5
3	How well do you feel that your immediate supervisor recognizes your potential?	Never Recognizes	Sometime Recognizes	Neutral	Recognizes	Fully Recognizes
		1	2	3	4	5
4	Regardless of how much formal authority your immediate supervisor has built into his or her position, what are the chances that he or she would be personally inclined to use power to help you solve problems in your work?	Never	Might Not	Neutral	Probably	Certainly
		1	2	3	4	5
5	Regardless of the amount of formal authority your immediate supervisor has, to what extent can you count on him or her to “bail you out” at his or her expense when you really need it.	Never	Might Not	Neutral	Probably	Certainly
		1	2	3	4	5
6	I have enough confidence in my immediate supervisor that I would defend and justify his or her decisions if he or she were not present to do so.	Never	Might Not	Neutral	Probably	Certainly
		1	2	3	4	5
7	How would you characterize your working relationship with your immediate supervisor?	Extremely Ineffective	Ineffective	Neutral	Effective	Extremely Effective
		1	2	3	4	5

## References

[1] Hofstede G. (1991). Empirical Models of Cultural Differences.

[2] Hofstede G. (2011). Dimensionalizing cultures: The Hofstede model in context. Int. J. Behav. Med..

[3] Islam N. (2004). Sifarish, Sycophants, Power and Collectivism: Administrative Culture in Pakistan. Int. Rev. Adm. Sci..

[4] Hofstede G. National Culture. https://hi.hofstede-insights.com/national-culture.

[5] Hofstede G. Country Comparison. https://www.hofstede-insights.com/country-comparison/pakistan/.

[6] Tang M., Hu W., Zhang H., Karwowski M., Kaufman J.C. (2017). Chapter 13—Creative Self-Efficacy from the Chinese Perspective: Review of Studies in Mainland China, Hong Kong, Taiwan, and Singapore. The Creative Self.

[7] Radda A.A., Majidadi M.A., Akanno S.N. (2015). Employee Engagement in Oil and Gas Sector. Studies.

[8] Mayer D.M., Aquino K., Greenbaum R.L., Kuenzi M. (2012). Who displays ethical leadership, and why does it matter? An examination of antecedents and consequences of ethical leadership. Acad. Manag. J..

[9] Grojean M.W., Resick C.J., Dickson M.W., Smith D.B. (2004). Leaders, values, and organizational climate: Examining leadership strategies for establishing an organizational climate regarding ethics. J. Bus. Ethics.

[10] Avey J.B., Wernsing T.S., Palanski M.E. (2012). Exploring the process of ethical leadership: The mediating role of employee voice and psychological ownership. J. Bus. Ethics.

[11] Cheng J.-W., Chang S.-C., Kuo J.-H., Cheung Y.-H. (2014). Ethical leadership, work engagement, and voice behavior. Ind. Manag. Data Syst..

[12] Sari U.T. (2019). The effect of ethical leadership on voice behavior: The role of mediators organizational identification and moderating self-efficacy for voice. J. Leadersh. Organ..

[13] Zheng Y., Epitropaki O., Graham L., Caveney N. (2021). Ethical Leadership and Ethical Voice: The Mediating Mechanisms of Value Internalization and Integrity Identity. J. Manag..

[14] De Hoogh A.H., Den Hartog D.N. (2008). Ethical and despotic leadership, relationships with leader’s social responsibility, top management team effectiveness and subordinates’ optimism: A multi-method study. Leadersh. Q..

[15] Milliken F.J., Morrison E.W., Hewlin P.F. (2003). An exploratory study of employee silence: Issues that employees don’t communicate upward and why. J. Manag. Stud..

[16] Pinder C.C., Harlos K.P. (2001). Employee silence: Quiescence and acquiescence as responses to perceived injustice. Research in Personnel and Human Resources Management.

[17] Sağnak M. (2017). Ethical leadership and teachers’ voice behavior: The mediating roles of ethical culture and psychological safety. Educ. Sci. Theory Pract..

[18] Walumbwa F.O., Schaubroeck J. (2009). Leader personality traits and employee voice behavior: Mediating roles of ethical leadership and work group psychological safety. J. Appl. Psychol..

[19] Graen G.B., Scandura T.A. (1987). Toward a psychology of dyadic organizing. Res. Organ. Behav..

[20] Walumbwa F.O., Mayer D.M., Wang P., Wang H., Workman K., Christensen A.L. (2011). Linking ethical leadership to employee performance: The roles of leader–member exchange, self-efficacy, and organizational identification. Organ. Behav. Hum. Decis. Processes.

[21] Eisenberger R., Cummings J., Armeli S., Lynch P. (1997). Perceived organizational support, discretionary treatment, and job satisfaction. J. Appl. Psychol..

[22] Kalyar M.N., Usta A., Shafique I. (2019). When ethical leadership and LMX are more effective in prompting creativity: The moderating role of psychological capital. Balt. J. Manag..

[23] Hussein H., Saade R. (2015). Employee voice in Business Management with a special reference to the Oil and Gas sector in UAE. Semant. Sch..

[24] Afkhami Ardakani M., Mehrabanfar E. (2015). Organizational silence, from roots to solutions: A case study in Iran petroleum industry. Iran. J. Oil Gas Sci. Technol..

[25] Fast N.J., Burris E.R., Bartel C.A. (2014). Managing to stay in the dark: Managerial self-efficacy, ego defensiveness, and the aversion to employee voice. Acad. Manag. J..

[26] Mordi C., Oruh E. A critical analysis of employee voice notion in Nigeria’s petroleum industry. Proceedings of the 31st Annual Conference of the British Academy of Management.

[27] Huang L., Paterson T.A. (2017). Group ethical voice: Influence of ethical leadership and impact on ethical performance. J. Manag..

[28] Klein K.J., Kozlowski S.W. (2000). Multilevel Theory, Research, and Methods in Organizations: Foundations, Extensions, and New Directions.

[29] LePine J.A., Van Dyne L. (2001). Voice and cooperative behavior as contrasting forms of contextual performance: Evidence of differential relationships with big five personality characteristics and cognitive ability. J. Appl. Psychol..

[30] Brown M.E., Treviño L.K., Harrison D.A. (2005). Ethical leadership: A social learning perspective for construct development and testing. Organ. Behav. Hum. Decis. Processes.

[31] Blau P.M. (1964). Power and Exchange in Social Life.

[32] Cropanzano R., Mitchell M.S. (2005). Social exchange theory: An interdisciplinary review. J. Manag..

[33] Bishop J.W., Scott K.D., Burroughs S.M. (2000). Support, commitment, and employee outcomes in a team environment. J. Manag..

[34] Edmondson A. (1999). Psychological safety and learning behavior in work teams. Adm. Sci. Q..

[35] Carmeli A. (2007). Social capital, psychological safety and learning behaviours from failure in organisations. Long Range Plan..

[36] Liu C. (2016). Does humble leadership behavior promote employees’ voice behavior—A dual mediating model. Open J. Bus. Manag..

[37] Yuan L., Vu M., Nguyen T. (2017). Linking ethical leadership to employee voice behavior: The role of leader–member exchange. Int. J. Bus. Manag. Stud..

[38] Newman A., Donohue R., Eva N. (2017). Psychological safety: A systematic review of the literature. Hum. Resour. Manag. Rev..

[39] Frazier M.L., Fainshmidt S., Klinger R.L., Pezeshkan A., Vracheva V. (2017). Psychological safety: A meta-analytic review and extension. Pers. Psychol..

[40] Yan A., Xiao Y. (2016). Servant leadership and employee voice behavior: A cross-level investigation in China. SpringerPlus.

[41] Graen G.B., Uhl-Bien M. (1995). Relationship-based approach to leadership: Development of leader–member exchange (LMX) theory of leadership over 25 years: Applying a multi-level multi-domain perspective. Leadersh. Q..

[42] Pfeffer J., Salancik G.R. (2003). The external control of organizations: A resource dependence perspective.

[43] French J.R., Raven B., Cartwright D. (1959). The bases of social power. Class. Organ. Theory.

[44] Dépret E., Fiske S.T. (1993). Social cognition and power: Some cognitive consequences of social structure as a source of control deprivation. Control Motivation and Social Cognition.

[45] Van Dyne L., LePine J.A. (1998). Helping and voice extra-role behaviors: Evidence of construct and predictive validity. Acad. Manag. J..

[46] Zeng J., Xu G. (2020). Linking ethical leadership to employee voice: The role of trust. Soc. Behav. Personal. Int. J..

[47] Jada U.R., Mukhopadhyay S., Titiyal R. (2019). Empowering leadership and innovative work behavior: A moderated mediation examination. J. Knowl. Manag..

[48] Hu Y., Zhu L., Zhou M., Li J., Maguire P., Sun H., Wang D. (2018). Exploring the influence of ethical leadership on voice behavior: How leader–member exchange, psychological safety and psychological empowerment influence employees’ willingness to speak out. Front. Psychol..

[49] Qi Y., Ming-Xia L. (2014). Ethical leadership, organizational identification and employee voice: Examining moderated mediation process in the Chinese insurance industry. Asia Pac. Bus. Rev..

[50] Chin T. (2013). How ethical leadership encourages employee voice behavior in China: The mediating role of organizational harmony. Int. Bus. Res..

[51] Pham V.-D. (2020). Ethical Leadership Supports Voice Behavior: Evidence from Vietnamese Service Firms. J. Bus. Econ. Dev..

[52] Nembhard I.M., Edmondson A.C. (2006). Making it safe: The effects of leader inclusiveness and professional status on psychological safety and improvement efforts in health care teams. J. Organ. Behav. Int. J. Ind. Occup. Organ. Psychol. Behav..

[53] Carmeli A., Tishler A., Edmondson A.C. (2012). CEO relational leadership and strategic decision quality in top management teams: The role of team trust and learning from failure. Strateg. Organ..

[54] Kahn W.A. (1990). Psychological Conditions of Personal Engagement and Disengagement at Work. Acad. Manag. J..

[55] Loi R., Lam L.W., Chan K.W. (2012). Coping with job insecurity: The role of procedural justice, ethical leadership and power distance orientation. J. Bus. Ethics.

[56] Talib M., Bibi Z., Zaman N.U. (2019). Mediating Effect of Psychological Safety on the relationship between Ethical Leadership and Employee’s Work Passion: Case Study of HEI’s of Baluchistan, Quetta. J. Manag. Sci..

[57] Tu Y., Lu X., Choi J.N., Guo W. (2019). Ethical leadership and team-level creativity: Mediation of psychological safety climate and moderation of supervisor support for creativity. J. Bus. Ethics.

[58] Edmondson A.C. (2003). Speaking up in the operating room: How team leaders promote learning in interdisciplinary action teams. J. Manag. Stud..

[59] Detert J.R., Burris E.R. (2007). Leadership behavior and employee voice: Is the door really open?. Acad. Manag. J..

[60] Liang J., Farh C.I.C., Farh J.-L. (2012). Psychological Antecedents of Promotive and Prohibitive Voice: A Two-Wave Examination. Acad. Manag. J..

[61] Zhao B., Olivera F. (2006). Error reporting in organizations. Acad. Manag. Rev..

[62] Ashford S.J., Rothbard N.P., Piderit S.K., Dutton J.E. (1998). Out on a limb: The role of context and impression management in selling gender-equity issues. Adm. Sci. Q..

[63] Bass B.M., Riggio R.E. Transformational Leadership.

[64] Jahanzeb S., Fatima T. (2018). How workplace ostracism influences interpersonal deviance: The mediating role of defensive silence and emotional exhaustion. J. Bus. Psychol..

[65] Mannan A., Kashif M. (2019). Being abused, dealt unfairly, and ethically conflicting? Quitting occupation in the lap of silence. Asia-Pac. J. Bus. Adm..

[66] Jorens E. Measurement Scale for Individual Psychological Safety; Erasmus University Thesis Repository: 2020. https://thesis.eur.nl/pub/52759.

[67] Xu M., Qin X., Dust S.B., DiRenzo M.S. (2019). Supervisor-subordinate proactive personality congruence and psychological safety: A signaling theory approach to employee voice behavior. Leadersh. Q..

[68] Uğurlu Ö.Y., Ayas S. (2016). The relationship between psychological safety and employee voice: The mediation role of affective commitment and intrinsic motivation. J. Bus. Res. Turk.

[69] Erkutlu H., Chafra J. (2015). Servant leadership and voice behavior in higher education. Hacet. Univ. J. Educ..

[70] Cheng J., Chang S., Kuo J., Lu K. (2014). Social relations and voice behavior: The mediating role of psychological safety. Criticism.

[71] Kark R., Carmeli A. (2009). Alive and creating: The mediating role of vitality and aliveness in the relationship between psychological safety and creative work involvement. J. Organ. Behav..

[72] Podsakoff P.M., MacKenzie S.B., Moorman R.H., Fetter R. (1990). Transformational leader behaviors and their effects on followers’ trust in leader, satisfaction, and organizational citizenship behaviors. Leadersh. Q..

[73] Jian L. (2014). Ethical Leadership and Employee Voice: Examining a Moderated-Mediation Model. Acta Psychol. Sin..

[74] Gerstner C.R., Day D.V. (1997). Meta-Analytic review of leader–member exchange theory: Correlates and construct issues. J. Appl. Psychol..

[75] Herdman A.O., Yang J., Arthur J.B. (2017). How does leader–member exchange disparity affect teamwork behavior and effectiveness in work groups? The moderating role of leader-leader exchange. J. Manag..

[76] Anand S., Hu J., Liden R.C., Vidyarthi P.R. (2011). Leader–member exchange: Recent research findings and prospects for the future. The Sage Handbook of Leadership.

[77] Martinaityte I., Sacramento C.A. (2013). When creativity enhances sales effectiveness: The moderating role of leader–member exchange. J. Organ. Behav..

[78] Sousa C.M., Coelho F., Guillamon-Saorin E. (2012). Personal Values, Autonomy, and Self-Efficacy: Evidence from frontline service employees. Int. J. Sel. Assess..

[79] Colella A., Varma A. (2001). The impact of subordinate disability on leader–member exchange relationships. Acad. Manag. J..

[80] Brown M.E., Mitchell M.S. (2010). Ethical and unethical leadership: Exploring new avenues for future research. Bus. Ethics Q..

[81] Botero I.C., Van Dyne L. (2009). Employee voice behavior: Interactive effects of LMX and power distance in the United States and Colombia. Manag. Commun. Q..

[82] Neubert M.J., Wu C., Roberts J.A. (2013). The influence of ethical leadership and regulatory focus on employee outcomes. Bus. Ethics Q..

[83] Zahra T.T. (2019). Impact of Ethical Leadership on Employee Voice Behavior and Innovative Work Behavior: Role of Psychological Empowerment, Leader–Member Exchange, Job Performance. Ph.D. Thesis.

[84] Nazir S., Shafi A., Asadullah M.A., Qun W., Khadim S. (2020). Linking paternalistic leadership to follower’s innovative work behavior: The influence of leader–member exchange and employee voice. Eur. J. Innov. Manag..

[85] Byun G., Dai Y., Lee S., Kang S. (2017). Leader trust, competence, LMX, and member performance: A moderated mediation framework. Psychol. Rep..

[86] Uhl-Bien M., Maslyn J.M. (2003). Reciprocity in manager-subordinate relationships: Components, configurations, and outcomes. J. Manag..

[87] Loi R., Chan K.W., Lam L.W. (2014). Leader–member exchange, organizational identification, and job satisfaction: A social identity perspective. J. Occup. Organ. Psychol..

[88] Opoku M.A., Choi S.B., Kang S.-W. (2020). Psychological safety in Ghana: Empirical analyses of antecedents and consequences. Int. J. Environ. Res. Public Health.

[89] Jin X., Jiang J., Li P., Li B. (2020). LMX and voice: A test in the Chinese hospitality industry. Anatolia.

[90] Niu W., Yuan Q., Qian S., Liu Z. (2018). Authentic leadership and employee job behaviors: The mediating role of relational and organizational identification and the moderating role of LMX. Curr. Psychol..

[91] The Institute of Chartered Accountants of Pakistan Oil & Gas Sector—Exploration, Production & Distribution: Surviving the Crisis & Entering the New Normal. https://www.icap.org.pk/paib/pdf/Oil&GasSector-Post-WebinarPaper.pdf.

[92] Wright P., Stern J., Phelan M. (2012). Core Psychiatry E-Book.

[93] Yamane T. (1965). Statistics: An introductory analysis. J. Am. Stat. Assoc..

[94] Scandura T.A., Graen G.B. (1984). Moderating effects of initial leader–member exchange status on the effects of a leadership intervention. J. Appl. Psychol..

[95] Field A., Miles J. (2009). Discovering Statistics Using SAS: And Sex and Drugs and Rock′n′Roll.

[96] Menard S. (1995). An introduction to logistic regression diagnostics. Applied Logistic Regression Analysis.

[97] Kock N. (2015). Common method bias in PLS-SEM: A full collinearity assessment approach. Int. J. e-Collab..

[98] Podsakoff P.M., MacKenzie S.B., Lee J.-Y., Podsakoff N.P. (2003). Common method biases in behavioral research: A critical review of the literature and recommended remedies. J. Appl. Psychol..

[99] Eichhorn B.R. (2014). Common Method Variance Techniques.

[100] Hair J., Black W.C., Babin B., Anderson R., Tatham R. (2014). Pearson new international edition. Multivariate Data Analysis.

[101] Fornell C., Larcker D.F. (1981). Evaluating structural equation models with unobservable variables and measurement error. J. Mark. Res..

[102] Enders C.K., Tofighi D. (2007). Centering predictor variables in cross-sectional multilevel models: A new look at an old issue. Psychol. Methods.

[103] Hayes A.F. (2017). Introduction to Mediation, Moderation, and Conditional Process Analysis: A Regression-Based Approach.

[104] Grant A.M. (2013). Rocking the boat but keeping it steady: The role of emotion regulation in employee voice. Acad. Manag. J..

[105] Edwards J.R., Lambert L.S. (2007). Methods for integrating moderation and mediation: A general analytical framework using moderated path analysis. Psychol. Methods.

[106] MacKinnon D.P., Lockwood C.M., Hoffman J.M., West S.G., Sheets V. (2002). A comparison of methods to test mediation and other intervening variable effects. Psychol. Methods.

[107] LePine J.A., Van Dyne L. (1998). Predicting voice behavior in work groups. J. Appl. Psychol..

[108] Hayduk L.A. (1987). Structural Equation Modeling with LISREL: Essentials and Advances.

[109] Baron R.M., Kenny D.A. (1986). The moderator–mediator variable distinction in social psychological research: Conceptual, strategic, and statistical considerations. J. Personal. Soc. Psychol..

[110] Dawson J.F. (2014). Moderation in management research: What, why, when, and how. J. Bus. Psychol..

[111] Cohen J., Cohen P., West S.G., Aiken L.S. (2003). Applied Multiple Regression/Correlation Analysis for the Behavioral Sciences.

[112] Schaubroeck J.M., Hannah S.T., Avolio B.J., Kozlowski S.W., Lord R.G., Treviño L.K., Dimotakis N., Peng A.C. (2012). Embedding ethical leadership within and across organization levels. Acad. Manag. J..

[113] Böckerman P., Bryson A., Ilmakunnas P. (2012). Does high involvement management improve worker wellbeing?. J. Econ. Behav. Organ..

[114] Thompson P.S., Bergeron D.M., Bolino M.C. (2020). No obligation? How gender influences the relationship between perceived organizational support and organizational citizenship behavior. J. Appl. Psychol..

